# The Medical Management of Insane Women

**Published:** 1864-11

**Authors:** Horatio Robinson Storer

**Affiliations:** Boston, Surgeon to the New England Hospital for Women


### THE MEDICAL MANAGEMENT OF INSANE WOMEN.

By HORATIO ROBINSON STORER, M.D., of Boston, Surgeon to the New England Hospital for Women.

Read before the Suffolk District Medical Society, October 1, 1864, and communicated for the Boston Medical and Surgical Journal.

II. Advisory Medical Boards requisite at Asylums.

In one of the April numbers of this Journal for the present year,* a series of papers was initiated upon the causation, nature and more rational treatment of insanity in women. My remarks were based, it was stated, upon extended observation, patient reflection, and a firm belief that the subject, though hitherto neglected, might be made to afford in practice important results. The few months that have since elapsed have been strongly confirmatory of every assumption then made; additional cases of the kind alluded to have been sent to me for examination and for treatment, and my views have been endorsed by gentlemen much older in the profession as in accordance with their own experience; and I acknowledge with pleasure the cordiality with which my attempt to elucidate a question among the most delicate and withal among the most important of those pertaining to obstetrics, has been so generally received.

The ground I have taken regarding this subject, is briefly the following:—

1. That the insanity of women is, in a large proportion of cases, of reflex character and origin.

* April 7, 1864, p. 189.

2. That in by far the majority of these, it is unattended by organic cerebral change; and therefore,

3. That like all other of the manifold reflex disturbances of women, it may be prevented, treated, cured by medical and surgical means.

Upon the above as a basis, I have now to offer certain practical suggestions concerning both the public and the private management of insane women. Of these, but a single one will be considered in the present communication.

To the point alluded to I have already called the attention of the profession at the late meeting of the American Medical Association, at New York; my remarks being embodied in a paper upon "The Relations of Female Patients to Hospitals for the Insane." This communication, having received the commendation of the Section of Practical Medicine and Obstetrics, was by it presented to the Association in Committee of the Whole, with the result of unanimous approval, the resolutions passed by the Association being as follows:—

"*Resolved*, That in the opinion of the American Medical Association, it is expedient that there should be attached to every public hospital for the Insane, one or more consulting physicians, who may be consulted at the discretion of the Superintendent; such measure being for the interest of the hospital, its medical officers, and its patients.

"*Resolved*, That a copy of the above resolution be transmitted to the Board of Trustees of each of our public hospitals for the Insane, and also to the Secretary of the Association of American Superintendents, with the request that it may be endorsed by that body, and the act proposed be urged upon the respective boards with which its members are officially connected."

It is the object of the present paper summarily to explain the advantages and necessity of the proposed measure, as comprising part of a systematic and more rational treatment of insane women; premising that with the single exception of Dr. Ray's most excellent institution at Providence, there is no asylum in this country provided with an advisory board. The so-called medical visitors attached to the Hartford Retreat are not appointed with reference to special professional consultation, while at the McLean Asylum, as is also the case at Bloomingdale, the consulting physicians and surgeons attached to the general hospital, have nothing whatever to do, officially, with the insane department. If any of these gentlemen have ever been called upon for advice, it has merely been as an act of courtesy and not as a right. They have not been selected with reference to any pre-eminence in this specialty, and they would probably none of them lay the slightest claim to be considered "expert" regarding insanity.

The advantages, as regards female patients, of an Advisory Board of Consulting Physicians at Insane Asylums would, I conceive, be several; applying—

I. To the Patient,
II. To the Hospital,
III. To the Superintendent,
IV. To the Profession, and
V. To the Community.

I—The benefit of an advisory board to patients will, in the case of females, be found to be extreme. My assumptions as to the nature and truly reflex character of the larger proportion of their psycho-pathological manifestations being granted, as must be done by every observer who has carefully studied these cases obstetrically (and this is the touchstone to which all the diseases of women must be subjected), it follows that, for the purposes of proper diagnonosis and of successful treatment, the usual measures resorted to at asylums in the case of insane women are wholly inefficient. To efface the effect in these cases present, we must reach its cause—persistent and active, in some instances even more active, after the world with its cares and its turmoil has been shut out. Seclusion does not restore a displaced womb; absence from friends, except in some cases from a husband, does not cure an inflamed or ulcerated cervix; the prohibition of letter-writing will not control suppression or redundance of the catamenia, nor a rigid diet the vagaries of an irritable ovary. What is required is that the case, whenever doubtful, should be carefully examined and thoroughly understood. This, as our asylums are at present conducted, and speaking in more than general terms, is never done with the female patients; it is not even attempted. The reasons of such seeming negligence I proceed to explain, and to suggest its cure.

The medical examination of an insane woman is not always easily made; and at an asylum is attended with certain risks to the medical attendant, existing in private practice with insane patients, unless due precautions are taken, but enhanced at asylums. I refer to the chance that the examination may appear to the patient's disordered mind a very different piece of business from what it is; and that it may be so reported to other patients and to her friends. Perhaps, indeed, the misconception may remain a sincere and persistent belief, even in case of eventual cure. These risks are in reality, as things now exist, far more tremendous than might be imagined; but when the charges are recollected that time and again have been made and pressed to trial in the courts by females whose delusions have been merely the effect of the transient administration of an anæsthetic,* it will be seen that with women who are really and permanently insane the danger is increased.

How can these difficulties, these risks, be avoided and overcome? So far as mere objections of the patient are concerned, or her forcible resistance even, etherization is sufficient, or merely the narcotization still so common for purely therapeutical ends in our asylums; but for protecting the medical officer's reputation and that of his hospital, no measure that has been or can be proposed will suffice, save an advisory board for medical consultation. I know that examinations may be attempted in the presence of junior medical officers or of a matron, but these are all parties of identical interest with the Superintendent; they are his subordinates, usually dependent for their places upon his good will, and therefore their presence and their evidence are without that moral weight which can alone be protective to such an examination as is required.

There has widely obtained an opinion, still existing to a certain extent as must be allowed by every candid observer, that asylums for the insane are rather houses of detention than hospital for cure; an idea based upon the prison treatment and discipline of these institutions in former days. Everything that can be done to disabuse the public of so great an error, or to prevent its having the slightest foundation in fact, is in every respect work well worthy our attention. For a large class of their patients, probably on the average fully one half, how can asylums be hospitals for cure, if the first step towards ascertaining the predisposing and exciting cause and true character of the mental disease is never taken? The apprehensions concerning the unnecessary or wrongful detention of female patients that have so often found vent in our journals and in the public courts, have generally been without reason or even show of reason as regards intentional wrong. Superintendents, there is every ground for believing, are men of honor, anxious to do their whole duty, and above fear or favor. But are there no reasons for fearing that in many, perhaps in all our asylums,

* For cases in point, that of Dr. Beale, of Philadelphia, and others, I refer to this Journal, November, 1858, p. 287; Philadelphia Medical Examiner, December, 1854; Western Law Monthly, April, 1860, p. 183; Wharton & Stilles's Medical Jurisprudence, 418, § 443.

female patients may occasionally be unintentionally detained, whose cases admit of relief or cure? The answer is evident enough from the statements already made, from the admissions of Superintendents themselves, from the results of similar cases in private practice to whom, unlike those in asylums, appropriate treatment has been applied, and from those instances of cure, however rare, that from time to time have spontaneously and accidentally occurred at asylums, where after many years, perhaps of no improvement at all, the grand climacteric has been passed, or a tumor become checked in its growth, or an old indolent pelvic abscess found vent, or the long-checked catamenial fountains again obtained for themselves effective discharge, and the mental disturbance that had been dependent upon the central lesion has disappeared. Such cases have been put upon record, but their true value and importance have not been recognized.

The public apprehensions to which I have alluded have some influence, how much or how little none of us can well appreciate, in preventing the sending of patients to insane asylums. Now it is conceded by all familiar with the subject, that the cure of every form of insanity, secondary and reflex, as well as primary and organic, depends in a great measure upon the earliness of the period at which proper treatment is commenced. Asylums are, or certainly should be, essentially hospitals for cure. Everything that we can do to make them so is a threefold advantage to the patient; it increases her chance of being sent at all to the institution by her friends, of being sent sufficiently early, and last, but not least important, of her being treated *secundum peritissimam artem*.

II.—I have said that by a consulting board taken from gentlemen in private practice, and therefore familiar with the details of obstetric or gynæcal management, these various difficulties as regards doing the best for the welfare of female patients would be likely to be overcome. Would it not also be of decided advantage and aid to the superintendent, looking at the question as it involves his best interests alone, and putting aside the satisfaction that would naturally accrue from the feeling that everything was being done for his patient that could be suggested by science or art? We are all of us familiar with the vexations to which of late years our own superintendents have been constantly subjected in courts of law. In the cases to which I refer, many gentlemen have been summoned from civil practice to give testimony for or against the managers of asylums. In a portion at least of these instances, there is reason to believe that there has existed one or another form of functional uterine or ovarian derangement which, had its existence and character been accurately determined, might of itself have thrown such light as satisfactorily to settle the question of sanity or insanity, of detention or non-detention, of permanent disability or of cure. In such a case were the disease to have been detected and properly treated, it ts probable that the delusions involved might have been made to permanently disappear, and an immense amount of scandal and professional disparagement avoided.

These are questions of direct interest to the profession as well as to any individual superintendent. Gentlemen have more than once acknowledged to me that they felt strongly tempted to retire from a position they had filled with usefulness and honor, merely because of the annoyance to which they were thus periodically subjected; and in case of a vacancy arising at any hospital, as has lately been the case at Northampton, it becomes a matter of vital importance to a candidate for him to decide whether he will voluntarily encounter the almost certain ordeal at law, to which he may be subjected by any one of the richer class of his female patients.

To superintendents the appointment of an advisory board would prove at once comfort, assistance, and safeguard. The probability of this was most courteously allowed by Drs. Tyler of the McLean Asylum, Walker, of South Boston, Jarvis, of Dorchester, and Choate, of Taunton, at the hearing granted the Commission in Insanity during the session of the Legislature of Massachusetts just ended; and its realization in practice was affirmed on the same occasion by Dr. Ray, of Rhode Island—the only superintendent in this country, from having an advisory board, placed in a position to express a competent opinion concerning his female patients. One of the members, indeed, of that very board, Dr. Mauran, of Providence, took occasion at a subsequent period, during the late session of the American Medical Association, at New York, to express in the most unqualified manner, from actual and extended experience, his approbation of the measure now proposed. Its necessity is indeed so palpable that we may well wonder that the change has not long since been effected. That it is still to be accomplished alone necessitates any extended argument.

I might easily adduce still further evidence of the character of that now given, but will content myself with a single line. In a letter dated August 3d ult., Dr. John S. Butler, the accomplished Superintendent of the Retreat at Hartford, writes me as follows, concerning a patient now under my charge:—"I always suspected that there might have been some uterine irritation or disorder acting perhaps as the exciting cause, but under all the embarrassing circumstances of our relative positions, I could not satisfy myself. Situated as I am here, I could not make such an examination as was necessary for a full understanding of her case; you can much better insist upon one than I could."

Could any testimony be more conclusive than this?

III.—Are there advantages to the profession from an advisory board, over and beyond those that I have spoken of as accruing to the superintendents themselves? Even here it must be allowed that there is an affirmative answer.

At the present time there are outside the asylums very few gentlemen skilled in psychiatry, very few even who lay claim to being psychical experts. The truth of both of these statements is evidenced at every important trial that involves questions of mental unsoundness. Few in private practice have opportunity of directly comparing, upon any large scale, the various phases of insanity; few, therefore, become directly and specially interested in their study. At the present time, so great is the scarcity of experts, that the resignation or death of the superintendent of any large asylum strkes with dismay the board of trustees of nearly every other similar institution in the country, lest the new incumbent to be chosen should be stolen from themselves. This is no exaggeration; the writer happens within the present season to have been semi-officially consulted concerning a vacancy in one of our asylums, and he knows very well what names were sent to him for the expression of his opinion, and the remarks concerning them that were made to him by trustees who possessed for the time being the services of the gentlemen referred to.

Now I contend that the establishment of advisory boards would not only tend to stimulate physicians to more frequent study of mental disease; it would afford a larger field from which to select gentlemen to fill the responsible positions of superintendents and trustees. It is very evident that such would necessarily be the case. No physician would be likely to be apointed to the advisory board who, with fair reputation in general practice, had not shown some interest, be it more or less, in the special department now under consideration, and had not a certain amount of familiarity with the innumerable mental vagaries of nervous women. Opportunities would of course, from his position, be likely to become frequent for further research, and the fascinations ever acknowledged to attend the study of mind diseased would tend to perfect him therein. As a single instance of what would naturally and generally be the result, it may be mentioned that shortly after the report of the late Massachusetts Commission in Insanity had been rendered, it was urged upon two of the three gentlemen constituting that board, that they should allow their own names to be used as eligible to the vacant superintendency of the Northampton Asylum; upon the third of their number similar arguments would have been used had he been a physician. It is unnecessary to add that in one at least of these instances, such position would have been incompatible with other though perhaps not higher aims; for I can conceive of no professional office with greater responsibilities, more constant call for all the eliments of one's better nature, more opportunity for making a deep and grand mark upon the tablet of the time, than the superintendency of one of our New England Hospitals for the Insane.

It may be alleged that there are few men fitted to be chosen as medical advisers to an asylum. This remark is undoubtedly true, but it depends entirely upon the profession how long such general incompetence shall exist. Already a door has been opened to the needed reform by the establishment of Dr. Tyler's class at the Harvard Medical School, an example soon to be followed, we trust, at all the colleges in the country. Towards such recognition of the claims of mental disease upon the profession at large, the measure I now propose would greatly tend, and on the other hand the more thoroughly mental disease becomes understood, the more incomprehensible it will appear that this greatest of elements towards the understanding and efficient treatment of female insanity should have been so utterly and persistently lost sight of.

V.—To the community at large, need I say, the reform proposed is of incalculable importance. As a mere matter of political economy, and to prove the worth in dollars and cents of every productive worker restored to reason, there were presented by the late Massachusetts Commission in its report to the Legislature some startling calculations and conclusions respecting the results attainable and already attained.* The statements to which I have referred, prepared with the minuteness and accuracy for which he is so celebrated, by Dr. Jarvis, of Dorchester, were presented more especially with reference to the male sex; but as regards the cost of support, and the pecuniary gain to friends and the State effected by cure, they equally apply to

* Mass. Senate Doc., 1864. No. 72, p. 5 et seq.

the case of women; while the chance of cure in women, provided only the proper treatment be afforded, is probably much greater than in men.

I have spoken of doubtful cases of insanity in women, which of late years have largely occupied the time of our courts and have kept the public mind excited and troubled. The community would be benefitted by the prevention of such trials or their more summary settlement, which can only be done by the advisory board now proposed.

I have referred to the danger of libellous charges against superintendents, in case they do their whole duty by the female patients entrusted to their charge. In the advisory board we have an efficient safeguard.

I have mentioned the increased confidence that would be felt in asylums should they be provided with such a board, and the larger proportion of female patients that would probably be sent for treatment at an earlier and more curable stage of the malady—in both respects a benefit being conferred upon the community—and the advantage that would accrue to it from a more general interest being taken in these diseases and greater proficiency in their treatment attained by medical men. Can we doubt that the board will soon be added to the management of every hospital?

In my communication upon this subject to the American Medical Association, I urged that the appointment should in every instance be an honorary one; as thereby tending to elevate the profession and the character of the services rendered. To my positions on this point, exceptions were taken by Dr. Griscom, of New York, upon the ground that physicians in private practice are at the best ill paid, and that by no one, more especially by the Association representing the profession at large, should action be taken tending to the establishment of further gratuitous labor.

While I strongly differed from the conclusions to which Dr. Griscom would press his argument, the resolutions originally offered to the Association were so far modified by passing this point in silence, as to leave it discretionary with trustees in each instance to pay or not to pay a salary to members of the advisory board; it will have been noticed that in this form the resolutions were unanimously passed.

In my own opinion, premising that the amendment of Dr. Griscom had been fully urged by him at the preliminary discussion before the Section of Practical Medicine and Obstetrics, and had been there rejected by a very large vote, the Section adopting and presenting the resolutions in the form first offered, as their own—in my opinion, I would still contend that these appointments should in every instance be strictly and purely honorary. I am led to this opinion by the following reasons:—

1st. Such appointments at ordinary general hospitals are always of this character; the only instance within my own personal knowledge in this country where consulting physicians or surgeons receive any emolument being at the General Hospital at Quebec—an example of short-sightedness on the part of its managers, or of parsimony on the part of its attendants only equalled by the whole detail of management of the Provincial Lunatic Hospital at Beauport, near that city. To the utter failure of this last-mentioned institution, the only one in the Lower Province, to accomplish its end, I referred in my remarks before the Association. It is a subject to which I may allude in an other communication, so necessary is it that the evil there existing should be exposed and abated.

If it is contrary to the ethics of the American Medical Association for physicians ever to give gratuitous advice in cases where a trifle could possibly be paid, then do we all of us who hold any honorary appointment at an hospital, daily and wittingly infringe the laws of that code; and we should all—among the number Dr. Griscom himself, one of the Physicians to the New York Hospital—be ignominiously expelled from the Association. That the gentleman referred to still holds his official position, and has not offered to resign it, is sufficient proof that his argument before the Association was so far fallacious as it was insufficient to control his own action in a matter similar to the case in hand.

2d. An honorary appointment is better for the interests of the hospital. Putting aside the fact that our public hospitals for the insane are in greater measure of a charitable character than remunerative, a large proportion of their patients being paupers, it is evident that the community, so sensative upon the point of possibly unnecessary detentions, would be more at ease with an unpaid than a paid advisory board; there would seem less likelihood of collusion, more probability that its action would be fair and unbiased.

3d. It is also better for the interests of the gentlemen composing the board. I acknowledge (as who does not?) the indirect advantages pertaining to an hospital appointment; that it is generally supposed to be conferred upon those by taste or previous education best fitted to fulfil its duties. But beyond these considerations, it is certain that the adviser who is unpaid at once holds a much more independent position, and that for this reason his dictum has much greater weight. Unpaid the position is much more likely to be unsought; and therefore, conferred, it gives the greater honor.

4th. And finally, an honorary appointment is also a better one for the community. Every salaried medical office is likely to become the object of contention and political intrigue, and therefore to be filled by incompetent persons. That such offices do from time to time happen to be occupied by the right men seems rather a matter of accident than of necessity, and in the nature of things it is hardly to be expected.

It should of course, as I have intimated, be left entirely to the discretion of the superintendent in all cases as to when and to what extent such a board as I have suggested should be consulted by him; the object being to offer him every opportunity of curing his patients, without in any way shackling his judgment, and above all to put it into his power to treat his female patients within the hospital as understandingly, as judiciously, and as thoroughly as the same women would be treated were they sane and at their homes.

The above remarks are commended alike to the attention of superintendents, of trustees, and of the profession at large. In my next communication upon the subject I shall enter into matters of stricter medical and surgical detail.

